# Modern contraceptive use, associated factors and reasons for non-use among young lactating mothers in the context of free family planning services in the Kaya municipality setting, Burkina Faso

**DOI:** 10.4102/jphia.v17i1.1661

**Published:** 2026-05-26

**Authors:** Abou Coulibaly, Adama Baguiya, Jeoffray Diendéré, Franck Garanet, Halima Tougri, Denise Kpebo, Seni Kouanda

**Affiliations:** 1 Department of Biomedical and Public Health, Institute of Research in Health Sciences, Ouagadougou, Burkina Faso; 2 African Institute of Public Health, Ouagadougou, Burkina Faso; 3 Maternal and Child Health Unit, National Public Health Institute, Abidjan, Côte d’Ivoire; 4 Department of Public Health, Medical School, University Allassane Ouattara, Abidjan, Côte d’Ivoire

**Keywords:** associated factors, free family planning services, modern contraceptive use, prevalence, young lactating mothers

## Abstract

**Background:**

Despite all the benefits of family planning (FP), African countries still have low contraceptive use and very high fertility rates.

**Aim:**

To measure modern contraceptive use among young lactating mothers in the context of the free contraceptive policy in the Kaya municipality setting.

**Setting:**

This study was conducted in the Kaya municipality, in the Koulsé region, Burkina Faso.

**Methods:**

This study is a secondary data analysis of the Kaya Demographic and Health Surveillance System data collected in the households between May 2022 and October 2022 (cross-sectional study). The dependent variable was the use of a modern contraceptive method (binary). Modified Poisson regression was used to identify associated factors.

**Results:**

The prevalence of modern contraceptive use among young lactating mothers was 53.9% (*n* = 536/995), with 41.4% using implants, followed by male condoms (38.2%), injectables (14.6%) and pills (5.4%). Modern contraceptive use was significantly higher among women who discussed FP with their spouses (adjusted prevalence ratios [aPR] = 1.4, 95% confidence interval [CI]: 1.2–1.8, *p* = 0.001) and among women who had ever used modern contraceptives before the last pregnancy (aPR = 1.9, 95% CI: 1.6–2.4, *p* < 0.001). Similarly, women in the rich quintile (aPR = 1.5, 95% CI: 1.1–2.0, *p* = 0.013) and the richest quintile (aPR = 1.5, 95% CI: 1.1–2.1, *p* = 0.014) were more likely to use modern contraceptives compared to the poorest.

**Conclusion:**

The prevalence of modern contraceptive use among young lactating mothers in Kaya is relatively high, with some modifiable factors that still explain contraceptive use.

**Contribution:**

This study documented evidence on contraceptive use among young lactating mothers under Burkina Faso’s free FP policy, highlighting equity gaps and actionable levers (communication between couples, prior exposure and men’s involvement).

## Introduction

Family planning (FP) refers to a couple’s decision to limit or space out the number of children they will have by using contraceptive methods. Contraceptive use has clear health benefits, as preventing unwanted pregnancies reduces the incidence of unsafe abortions and thus saves lives, because abortions are responsible for 7% – 9% of maternal deaths.^1^ Some studies have also shown that FP helps to reduce child mortality because contraception, by lengthening the birth interval, prevents pregnancies and closely spaced, premature births, which are significant predictors of child mortality.^2^

Worldwide, among 1.9 billion women of reproductive age (15–49 years), an estimated 874 million women use a modern contraceptive method. Although substantial progress has been made, there are still 164 million women who want to delay or avoid pregnancy and are not using any contraceptive method and thus are considered to have an unmet need for FP.^2^

Despite all the benefits of FP, African countries still have low contraceptive use and very high fertility rates. The World Bank identified 10 countries in sub-Saharan Africa with fertility rates above five in 2021.^3^ While FP is essential throughout the reproductive lives of individuals and couples, postpartum family planning (PPFP) focuses on preventing unwanted or closely spaced pregnancies in the months following childbirth. Before and during this postpartum period, there are many contacts with health services that provide an opportunity to offer women who have recently given birth with appropriate counselling and to promote contraceptive use. These opportunities include antenatal care (ANC) appointments, delivery and the immediate postpartum period, as well as postpartum consultations at 6 days and 6 weeks, healthy infant consultations, infant immunisation sessions and consultations for maternal or infant health problems. Postpartum family planning is crucial in addressing unmet needs for FP, as postpartum women are among those most in need of these services. In addition, closely spaced pregnancies in the first year after childbirth are the most dangerous for both mother and child, as they carry an increased risk of adverse outcomes such as prematurity, low birth weight and intrauterine growth restriction.^4^

Africa has the highest fertility rate in the world, and almost a quarter of women of reproductive age – nearly 47 million – still have an unmet need for contraception.^5^ Several interventions have been tested to overcome financial barriers and increase women’s access to contraceptive methods (e.g. cash transfer programmes, distribution of free vouchers and community or performance-based financing, free FP, etc.).^6,7,8^ Burkina Faso was one of the first countries in sub-Saharan Africa to adopt a free FP policy, partly justified by the success of the national FP weeks organised each year.^9^ The free policy, which was piloted in June 2019 and scaled up in July 2020, covers all sexually active people and the cost of long-acting and short-acting contraceptive methods, outpatient curative care to manage unwanted side effects and inpatient curative care in the event of complications. Six months after the implementation of the free policy, a study by Tiendrebeogo et al.^10^ showed that the abolition of user fees for FP led to an 86% increase in the likelihood of contraceptive use and a 13-point reduction in the prevalence of unmet need for contraception.

The non-use of modern contraceptives among young lactating mothers may cause several health problems if they become pregnant. Indeed, this group of women is exposed to the potential risk of closely spaced pregnancies, with all the possible severe complications.^11,12^ This means that the non-use of contraception exposes them to a new pregnancy with a birth interval of less than 33 months, which is below the World Health Organization recommendation of at least 24 months to avoid a very high risk of morbidity and mortality for the woman and her child.^11,12^

Therefore, firstly, the rationale for conducting this study among breastfeeding women is that, to the best of our knowledge, since the implementation of free FP in Burkina Faso, studies measuring contraceptive use have targeted all married and unmarried women, irrespective of their lactating status.^13,14^ Few studies have examined contraceptive use specifically among young lactating mothers under the new free FP policy in Burkina Faso. Secondly, given that one of the reasons for not using contraception is the desire to have a child, this paper aims to examine the modern contraceptive use and factors associated with it among breastfeeding women with a child under 24 months in a semi-rural setting of Burkina Faso. Given that they have young children, they are unlikely to have another child at the time of the study.

## Research methods and design

### Study setting

The study was conducted in the Kaya Health and Demographic Surveillance System (Kaya-HDSS) area, located in the Kaya municipality, Koulsé region, Burkina Faso. It has been operational since 2007. According to the latest general population and housing census, the population of Kaya was estimated to be 208 682 in 2019.^15^ In 2022, the resident population of the site catchment area was 82 723. It has one medical centre, six primary health centres, six rural health centres and a few private health centres. In addition to these primary health centres, the observatory area also includes the regional hospital, which provides specialised care and is a referral centre for health centres in the four provinces of the Koulsé region. The Kaya-HDSS aims to study demographic, infectious and chronic disease indicators in the district, monitor changes in health over time, evaluate health programmes and provide a basis for policy decisions and the capacity to improve the community’s health. The site collects maternal and reproductive health data annually, including FP, child health and many other topics.^16^

### Operational definitions

Modern contraceptive methods include female and male sterilisation, intrauterine devices (IUDs), injectables, implants, pills and condoms.^17^

### Study population

The study population consisted of women aged below 25 years residing in the Kaya municipality who were breastfeeding a child below 24 months at the time of data collection.

### Study design and period

This was a secondary analysis of data from the Kaya-HDSS, collected cross-sectionally in households between May 2022 and October 2022.

### Data source and data collection techniques

Interviewers collected data using questionnaires administered to all women in the households during their visits. Women’s socio-demographic characteristics were collected through direct interviews, and certain variables were extracted from their health records.

### Variables

#### Dependent variable

The dependent variable in our study was the use of a modern contraceptive method. It was a binary variable, with the value ‘1’ for women who were currently using a modern contraceptive method at the time of data collection and ‘0’ otherwise. Modern contraceptive methods include female and male sterilisation, IUDs, injectables, implants, pills and condoms.^17^

#### Independent variables

The independent variables used in this study were socio-demographic variables (age, occupation, wealth quintile, marital status, education level and place of residence) and variables related to FP (receipt of counselling, need for future counselling and discussion with spouse or partner about FP), as detailed in Table 1.

**TABLE 1:** Independent variables.

Group of variables	Variables	Categories
Women’s characteristics	Age groups (in years)	Under 20 years, 20–24 years
	Education level	None, primary, secondary and above
	Occupation	Housewife, agriculture, sales or other income generating activities, pupil or student or civil servant
	Marital status	Single or separated, monogamous union, polygamous union
	Location	Urban, rural
	Wealth quintiles	Poorest, poor, middle, rich, richest
	Relationship to head of household	Head of household, wife of head of household, daughter of head of household, other relationship
	Number of alive children	1, 2, 3 and +
	Had a dead child	No, Yes
	Had a previous abortion	No, Yes
Family planning characteristics	Would you like advice on family planning?	No, Yes
	Have you ever received advice and information on family planning?	No, Yes
	Ever discussed with a partner about family planning	No, Yes
	Ever used a modern contraceptive before the last pregnancy	No, Yes

### Data analysis

Qualitative variables were described using frequencies. A modified Poisson regression was used to identify factors associated with modern contraceptive use. Associations were estimated using adjusted prevalence ratio (aPR) with 95% confidence interval (CI). The selection of independent variables and their inclusion in the final model were based on the variables in the database and the literature review conducted for this purpose. The model’s goodness of fit was assessed using the Akaike Information Criterion (AIC), with a lower AIC indicating a better fit. All analyses were performed using Stata version 18, with the significance level set at *p* < 0.05.

### Ethical considerations

An application for full ethical approval was made to the Burkina Faso Health Research Ethics Committee, and ethics consent was received on 07 July 2021 for the research protocol (ethical research number: 2021-07-165). All procedures performed in studies involving human participants were in accordance with the ethical standards of the institutional and national research committee and with the 1964 Helsinki Declaration and its later amendments or comparable ethical standards.

Written informed consent was obtained from all individual participants involved in the study. Women’s anonymity and confidentiality were maintained throughout the data collection and processing (by separating identifying information from the data, using security measures to protect the data and limiting access to it; identifying information, when collected, was protected by using codes instead of names (ID variable) and storing identifying information separately and securely).

## Results

**FIGURE 1:**
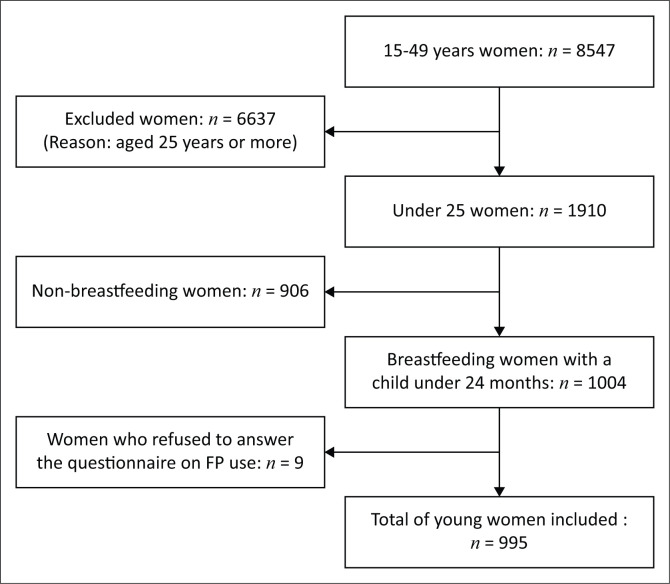
Study flowchart.

Our study included 995 young lactating mothers. Figure 1 shows the recruitment process for the target population.

### Socio-demographic characteristics of the women (*N* = 995)

Most women were in a monogamous union (78.6%). Almost half had no education (45.4%) and were housewives (69.2%). These results are presented in Table 2.

**TABLE 2:** Socio-demographic characteristics of women (*N* = 995).

Characteristics	*n*	%
**Age (years)**		
Under 20	184	18.5
20–24	811	81.5
**Marital status (*n* = 942)**		
Single or separated	90	9.6
Monogamous union	740	78.6
Polygamous union	112	11.9
**Wealth quintiles (*n* = 937)**		
Poorest	191	20.4
Poor	211	22.5
Middle	217	23.2
Rich	190	20.3
Richest	128	13.7
**Education level**		
None	452	45.4
Primary	128	12.9
Secondary or tertiary	415	41.7
**Occupation (*n* = 944)**		
Housewife	653	69.2
Agriculture	127	13.5
Sales or other income-generating activities	89	9.4
Pupil or student or civil servant	75	7.9
**Relationship to head of household**		
Head of household	34	3.4
Wife of the head of household	841	84.5
Daughter of the head of household	62	6.2
Other relationship	58	5.8
**Residence**		
Urban	596	59.9
Rural	399	40.1
**Number of alive children (*n* = 993)**		
1	595	59.9
2	318	32.0
3 and +	80	8.1
**Had a dead child**		
No	978	98.3
Yes	17	1.7
**Had a previous abortion**		
No	971	97.6
Yes	24	2.4

### Family planning characteristics

Almost all women (92.6%) reported that they needed information and advice on contraceptive methods. However, 87.6% reported that they had already received FP information and advice. More than half of the women (53.3%) had never discussed FP with their spouse or partners, and 49.6% had ever used a modern contraceptive before the last pregnancy. These results are presented in Table 3.

**TABLE 3:** Family planning characteristics (*N* = 995).

Family planning characteristics	*n*	%
**Would you like advice on family planning?**		
No	74	7.4
Yes	921	92.6
**Have you ever received advice and information on family planning?**		
No	123	12.4
Yes	872	87.6
**Ever discussed with a partner about family planning**		
No	528	53.3
Yes	462	46.7
**Ever used a modern contraceptive before the last pregnancy**		
No	501	50.4
Yes	494	49.6

### Prevalence of modern contraceptive use

In this study, 536 out of 995 women were using a modern contraceptive method, giving a prevalence of 53.9% (95% CI: 50.8–57.0). Two hundred and twenty-two (*n* = 222) women were implant users (41.4%), 78 were injectable users (14.6%), 29 were pill users (5.4%) and 10 women were lactational amenorrhoea method users (1.9%). The other methods, IUD, female condom, calendar method and abstinence, were each used by only one woman. For the male condom, 205 women among those who were not using any modern contraceptive method (38.2%) reported that their spouses or partners were users, with 35 women reporting systematic use and 170 women reporting occasional use. Among those who were using a modern contraceptive method, 94 women reported that their spouses or partners were condom users (dual contraceptive users).

Looking at the different characteristics, we found that the use of modern contraceptives varied according to socio-economic level (wealth quintiles), women’s education, place of residence, need for FP advice, receipt of FP advice and information during the last pregnancy, prior contraceptive use and discussions with spouses or partners about FP. These findings are shown in Table 4.

**TABLE 4:** Use of modern contraceptives by characteristics.

Women’s characteristics	Total (*n*)	Modern contraceptive users		*p*-value
		*n*	%	
**Age (years)**	-	-	-	0.424
Under 20	184	104	56.5	-
20–24	811	432	53.3	-
**Marital status (*n* = 942)**	-	-	-	0.089
Single or separated	90	52	57.8	-
Monogamous union	740	408	55.1	-
Polygamous union	112	50	44.6	-
**Wealth quintiles (*n* = 937)**	-	-	-	< 0.001
Poorest	191	75	39.3	-
Poor	211	108	51.2	-
Middle	217	113	52.1	-
Rich	190	120	63.2	-
Richest	128	89	69.5	-
**Education level**	-	-	-	0.004
None	452	218	48.2	-
Primary	128	72	56.2	-
Secondary or tertiary	415	246	59.3	-
**Occupation (*n* = 944)**	-	-	-	0.111
Housewife	653	362	55.4	-
Agriculture	127	57	44.9	-
Sales or other income-generating activities	89	53	59.6	-
Pupil or student or civil servant	75	39	52.0	-
**Relationship to head of household**	-	-	-	0.063
Head of household	34	12	35.3	-
Wife of the head of household	841	465	55.3	-
Daughter of the head of household	62	28	45.2	-
Other relationship	58	31	53.4	-
**Residence**	-	-	-	< 0.001
Urban	596	364	61.1	-
Rural	399	172	43.1	-
**Number of children alive (*n* = 993)**	-	-	-	0.727
1	595	327	55.0	-
2	318	168	52.8	-
3 and +	80	41	51.2	-
**Had a dead child**	-	-	-	0.570
No	978	528	54.0	-
Yes	17	8	47.1	-
**Had a previous abortion**	-	-	-	0.657
No	971	522	53.8	-
Yes	24	14	58.3	-
**Would you like advice on family planning?**				< 0.001
No	74	19	25.7	-
Yes	921	517	56.1	-
**Have you ever received advice and information on family planning?**	-	-	-	< 0.001
No	123	43	35.0	-
Yes	872	493	56.5	-
**Ever discussed with partner about family planning**	-	-	-	< 0.001
No	528	196	37.1	-
Yes	462	335	72.5	-
**Ever used a modern contraceptive before the last pregnancy**	-	-	-	< 0.001
No	501	160	31.9	-
Yes	494	376	76.1	-

### Reasons for not using modern contraceptives

**FIGURE 2:**
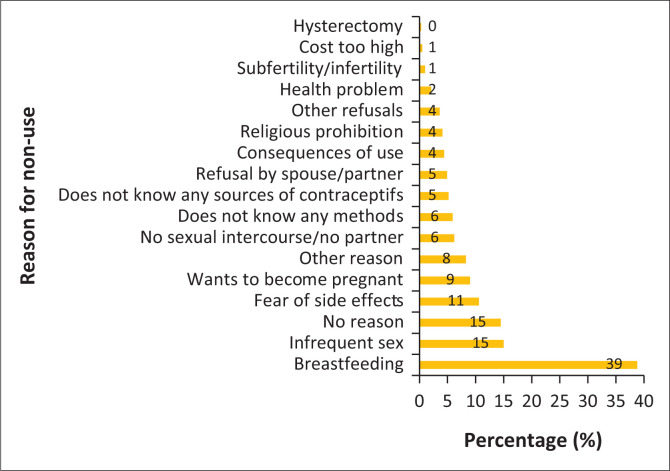
Reasons for not using modern contraceptives among young lactating mothers.

The 387 women who did not use modern contraceptives most frequently cited breastfeeding (39%) and fear of side effects (11%) as reasons for not using. However, 15% of women did not give any reason for non-use. Opposition from a spouse or partner and religious beliefs were also reasons given by women. The detailed reasons are shown in Figure 2.

### Factors associated with modern contraceptive use

Modern contraceptive use was significantly higher among women who discussed FP with their spouses (aPR = 1.4, 95% CI: 1.2–1.8, *p* = 0.001) compared with those who did not discuss FP with their partner and among women who had ever used a modern contraceptive before the last pregnancy (aPR = 1.9, 95% CI: 1.6–2.4, *p* < 0.001) compared with those who had never used a modern contraceptive before the last pregnancy. Similarly, women in the rich quintile (aPR = 1.5, 95% CI: 1.1–2.0, *p* = 0.013) and the richest quintile (aPR = 1.5, 95% CI: 1.1–2.1, *p* = 0.014) were more likely to use modern contraceptives compared to the poorest quintile. Certain variables, such as place of residence, need for FP advice, receipt of FP advice and information during the last pregnancy, although significantly associated with contraceptive use in univariate analysis, were removed during the multivariate analysis because their inclusion did not improve the adjusted model. These results are presented in Table 5.

**TABLE 5:** Factors associated with contraceptive use in the multivariate analysis.

Characteristics	Crude prevalence ratio		*p*-value	Adjusted prevalence ratio		*p*-value
	aPR	95% CI		aPR	95% CI	
**Ever discussed with a partner about family planning**						
No	Ref	Ref	-	Ref	Ref	-
Yes	2.0	1.6–2.3	< 0.001	1.4	1.2–1.8	0.001
**Ever used a modern contraceptive before the last pregnancy**						
No	Ref	Ref	-	Ref	Ref	-
Yes	2.4	2.0–2.9	< 0.001	1.9	1.6–2.4	< 0.001
**Would you like advice on family planning?**						
No	Ref	Ref	-	Ref	Ref	-
Yes	2.2	1.4–3.5	0.001	1.8	1.0–3.2	0.055
**Have you ever received advice and information on family planning?**						
No	Ref	Ref	-	Ref	Ref	-
Yes	1.6	1.2–2.2	0.003	0.8	0.6–1.2	0.383
**Wealth quintiles**						
Poorest	Ref	Ref	-	Ref	Ref	-
Poor	1.3	1.0–1.8	0.078	1.3	0.9–1.7	0.138
Middle	1.3	1.0–1.8	0.058	1.3	0.9–1.7	0.128
Rich	1.6	1.2–2.1	0.001	1.5	1.1–2.0	0.013
Richest	1.8	1.3–2.4	< 0.001	1.5	1.1–2.1	0.014
**Education level**						
None	Ref	Ref	-	Ref	Ref	-
Primary	1.2	0.9–1.5	0.258	1.1	0.8–1.5	0.439
Secondary or tertiary	1.2	1.0–1.5	0.027	1.1	0.9–1.4	0.289
**Age (years)**						
Under 20	Ref	Ref	-	-	-	-
20–24	0.9	0.8–1.2	0.587	-	-	-
**Marital status**						
Single or separated	Ref	Ref	-	-	-	-
Monogamous union	1.0	0.7–1.3	0.751	-	-	-
Polygamous union	0.8	0.5–1.1	0.193	-	-	-
**Occupation**						
Housewife	Ref	Ref	-	-	-	-
Agriculture	1.1	0.8–1.5	0.704	-	-	-
Sales or other income-generating activities	0.9	0.6–1.3	0.479	-	-	-
Pupil or student or civil servant	1.1	0.8–1.7	0.520	-	-	-
**Relationship to head of household**						
Head of household	Ref	Ref	-	-	-	-
Wife of the head of household	1.6	0.9–2.8	0.125	-	-	-
Daughter of the head of household	1.3	0.7–2.5	0.475	-	-	-
Other relationship	1.5	0.8–2.9	0.222	-	-	-
**Residence**						
Urban	Ref	Ref	-	-	-	-
Rural	0.7	0.6–0.8	< 0.001	-	-	-
**Number of alive children**						
1	Ref	Ref	-	-	-	-
2	1.0	0.8–1.2	0.677	-	-	-
3 and +	0.9	0.7–1.3	0.673	-	-	-
**Had a dead child**						
No	Ref	Ref	-	-	-	-
Yes	0.9	0.4–1.8	0.700	-	-	-
**Had a previous abortion**						
No	Ref	Ref	-	-	-	-
Yes	1.1	0.6–1.8	0.763	-	-	-

aPR, adjusted prevalence ratio; CI, confidence interval; Ref, reference.

## Discussion

We identified the prevalence and factors associated with modern contraceptive use among young lactating mothers in the Kaya municipality. Overall, the prevalence of modern contraceptive use among young lactating mothers was 53.9%. This study showed that discussions within couples influenced the use of FP. It also showed that women who had used contraception before the last pregnancy used it more than those who did not. Finally, it showed that women with a higher socio-economic status used modern contraceptives more than the poorest women.

The prevalence of modern contraceptive use among young lactating women in this study was relatively high. This could be because of the national policy of free FP services, which has been in place in Burkina Faso since July 2020 in the Koulsé region, where the city of Kaya is located. Financial barriers to accessing contraception may have been reduced with free FP and may therefore have led to increased use of contraceptive methods.

In our study, condoms remained the second most commonly used contraceptive method (38.2%) after implants (41.4%). A similar result was reported by Ahissou et al. in Benin, who noted that male condoms were one of the most commonly used methods among young women.^18^ It is therefore important that information sessions be held for both women and men to ensure that the rules surrounding condom use are followed. Even among regular condom users, there may be cases of incorrect use, such as genital contact before or after condom use, as well as cases of breakage, as previously reported by some authors. In fact, a published study showed that the prevalence of incorrect condom use can reach 40% – 50% among condom users.^19^ This calls for in-depth awareness raising on the importance of using condoms correctly and thus avoiding closely spaced pregnancies in breastfeeding women.

Findings on modifiable determinants are particularly important because they could inform the design of programmes and interventions. Our results showed that one of the major determinants of current method use was whether the woman had already used at least one modern contraceptive method. Indeed, the experience can increase women’s knowledge, encouraging them to use contraceptive methods more regularly. This result indicates that the most crucial step is convincing women to adopt a method for the first time. This may be one of the best ways to combat fears about side effects. Previous studies have already shown that the experience of contraceptive use is associated with the reuse of contraceptive methods. For example, Saizonou et al. in Benin found an increased likelihood of contraceptive use among participants who had already used a contraceptive.^20^ Previous use of contraceptive methods can also help women to better understand side effects and stimulate the search for more proactive advice to manage them, ensuring continued use and satisfaction with a new method. Programmes should be designed to provide guidance tailored to correct and consistent use, based on a person’s past challenges. Indeed, understanding past experiences can not only inform the design of planning programmes to ensure that a full range of methods are available and accessible, but it can also prompt a review of the way counselling is delivered to health service users. For example, counselling sessions may specifically target the reasons for changing or discontinuing a method or dissatisfaction with previous use, proactively addressing these barriers, such as side effects, to improve long-term use and continuity of use.

In addition, the study showed that there is still a significant difference in the likelihood of using a modern contraceptive method between rich and poor women, even though contraceptives are currently accessible in Burkina Faso. It may be that people are not sufficiently aware of this measure and therefore do not use the free methods. Although the study by Tiendrebeogo et al.,^10^ conducted in Burkina Faso 6 months after the implementation of the free policy, supports this hypothesis, it is less likely because free FP services were expanded in July 2020, almost 4 years after implementation. Indeed, Tiendrebeogo et al. showed that, of the 1471 women of reproductive age surveyed 6 months after the abolition of user fees for FP services, 56% were aware of the policy, and knowledge of the fee abolition policy was associated with a 46% increase in the likelihood of contraceptive use among women aged 15–49 years. Most likely, the policy of free contraception has limits in its application, with residual expenditure by women on contraception, thus creating disparities between rich and poor women. The findings of Browne et al. also support this.^21^ These authors reported, in their evaluation of the free contraceptive policy in Burkina Faso 6 months after its introduction, that barriers to implementation included inadequate communication about the policy, shortages of supplies and contraceptive methods and delays in government reimbursement. Indeed, in their study, 39% of participants continued to pay for FP services despite the new policy, mainly because of stock-outs that forced them to buy their contraceptives elsewhere. These findings may explain the persistent difference in contraceptive use between rich and poor women. Furthermore, there could be differential information needs between the richest and poorest quintiles. For example, the rich may require more specific information, while women in low-income households may need programmes that simultaneously address financial barriers and stigma through targeted awareness-raising, accessible services and culturally appropriate communication.

Finally, discussions within couples have a significant impact on women’s contraceptive use. Many authors have reported this in different contexts. For example, a systematic review of 14 articles on the role of men in the uptake of contraceptive methods reported a highly significant association between non-opposition by partners and a higher likelihood of contraceptive use among women.^22^ Another study showed that approximately 59.2% of couples who had discussed FP in the 12 months prior to the survey date were using contraceptives, while only 39.2% of couples who had not discussed it were using contraceptives.^23^ In Niger, similar results were also found.^24^ Efforts should be made to better communicate the fact that FP is a woman’s right, on the one hand, and also to involve men more in sexual and reproductive health policies, so as to improve their knowledge of FP and promote discussion within couples. Indeed, these results provide data to target interventions that facilitate joint decision-making, including the use of mass media to encourage private discussions within couples about FP. Also, it may be important to offer, for example, counselling to couples (both partners together) rather than focusing only on women or men, as already recommended by Fantaye et al. in Ethiopia.^25^ This includes giving both partners the opportunity to learn together about the different methods and goals of FP. This can include group sessions, home visits or other community-based approaches that bring couples together and can be particularly important in African contexts where traditional gender roles can impede communication.

This study found that a significant proportion of women did not use modern contraceptives because of side effects. This finding has been reported in previous studies. Indeed, a literature review of factors influencing contraceptive use in sub-Saharan Africa between 2005 and 2015, published in 2017, found that the negative factors reducing contraceptive use were women’s misconceptions about the side effects of contraception.^26^ Awareness campaigns should be conducted to minimise the extent of these misconceptions about contraception. Our results also showed that men and other family members are reluctant to use modern contraceptives by lactating women (5% for men’s refusal and 4% for other family members’ refusal). Several studies have found similar results.^27,28^ It is therefore necessary to develop awareness-raising and information campaigns aimed at men so that they accept their wives’ use of contraceptive methods.

### Strengths and limitations

This study has several limitations. Firstly, the cross-sectional design prevents us from establishing a causal link between the dependent variable and the predictors. Secondly, the study population is restricted to young lactating mothers. Although the results can be generalised to all young lactating mothers in the Kaya municipality, they are not generalisable to all women in the region or nationwide. Thirdly, contraceptive use was measured based on women’s self-reports, which may not always accurately reflect reality. Fourthly, because this study used secondary data, some missing variables may explain contraceptive use among young lactating mothers. Nonetheless, the strength of this study is that we did not select women; instead, we interviewed all women within the area covered by the Kaya-HDSS.

## Conclusion

The prevalence of modern contraceptive use among young lactating mothers in the Kaya municipality, Koulsé, Burkina Faso, is relatively high. As our study population consists of young lactating mothers, the use of modern contraceptives among these women should be higher because they are at risk of having a short birth interval, with its consequences for women’s health and pregnancy outcomes, should they become pregnant. It is, therefore, necessary to continue to raise awareness among women and men in general and young lactating mothers in particular, so that they adopt more modern contraceptive methods to improve their health and well-being. These awareness-raising campaigns and interventions should focus on strengthening counselling for lactating mothers at every contact with health centres, improving free access policies by fully removing financial barriers to accessing FP services and involving men.
